# Hydrogen sulfide metabolism regulates endothelial solute barrier function

**DOI:** 10.1016/j.redox.2016.08.004

**Published:** 2016-08-11

**Authors:** Shuai Yuan, Sibile Pardue, Xinggui Shen, J. Steven Alexander, A. Wayne Orr, Christopher G. Kevil

**Affiliations:** a Department of Cellular Biology and Anatomy, Louisiana State University Health Sciences Center, Shreveport, LA 71103, USA; b Department of Pathology, Louisiana State University Health Sciences Center, Shreveport, LA 71103, USA; c Department of Molecular and Cellular Physiology, Louisiana State University Health Sciences Center, Shreveport, LA 71103, USA; d Center for Cardiovascular Diseases and Sciences, Louisiana State University Health Sciences Center, Shreveport, LA 71103, USA

**Keywords:** Hydrogen sulfide, Cystathionine γ-lyase, Endothelial permeability, Polysulfide

## Abstract

Hydrogen sulfide (H_2_S) is an important gaseous signaling molecule in the cardiovascular system. In addition to free H_2_S, H_2_S can be oxidized to polysulfide which can be biologically active. Since the impact of H_2_S on endothelial solute barrier function is not known, we sought to determine whether H_2_S and its various metabolites affect endothelial permeability. In vitro permeability was evaluated using albumin flux and transendothelial electrical resistance. Different H_2_S donors were used to examine the effects of exogenous H_2_S. To evaluate the role of endogenous H_2_S, mouse aortic endothelial cells (MAECs) were isolated from wild type mice and mice lacking cystathionine γ-lyase (CSE), a predominant source of H_2_S in endothelial cells. In vivo permeability was evaluated using the Miles assay. We observed that polysulfide donors induced rapid albumin flux across endothelium. Comparatively, free sulfide donors increased permeability only with higher concentrations and at later time points. Increased solute permeability was associated with disruption of endothelial junction proteins claudin 5 and VE-cadherin, along with enhanced actin stress fiber formation. Importantly, sulfide donors that increase permeability elicited a preferential increase in polysulfide levels within endothelium. Similarly, CSE deficient MAECs showed enhanced solute barrier function along with reduced endogenous bound sulfane sulfur. CSE siRNA knockdown also enhanced endothelial junction structures with increased claudin 5 protein expression. In vivo, CSE genetic deficiency significantly blunted VEGF induced hyperpermeability revealing an important role of the enzyme for barrier function. In summary, endothelial solute permeability is critically regulated via exogenous and endogenous sulfide bioavailability with a prominent role of polysulfides.

## Highlights

• Polysulfide from cystathionine γ-lyase (CSE) and exogenous polysulfide donors increases endothelial permeability.
• The ability of polysulfide to increase permeability is associated with junction disruption and stress fiber formation.
• CSE expression in vivo regulates VEGF induced hyper-permeability.

## Graphical abstract

**Figure:**
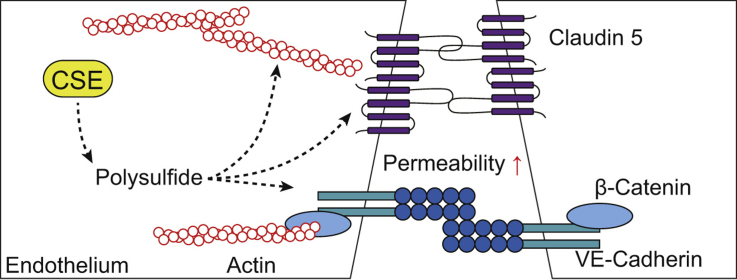

## 1 Introduction

Endothelial cells form the innermost layer of blood vessels and serve as a barrier confining blood within the vessel. This barrier function exhibits heterogeneity across different vascular beds to meet local tissue microenvironments and functions [3]. Endothelial barriers in large to medium vessels throughout the body are considered to be largely impermeable to various molecules in the blood under physiological conditions. In contrast, as the major exchange vessels, capillaries witness continuous exchange of soluble contents between blood and the interstitial tissue. On the other hand, permeability can be induced in other vascular beds (predominantly in the post-capillary vanules) where basal permeability is low. Increased permeability is an essential part of acute and chronic inflammation and is critical for compartmentalization and the recruitment of inflammatory cells. Increased permeability may also favor angiogenesis and tissue repair by changing extracellular matrix composition and obliterate contact inhibition between cells [2], [15]. Therefore, endothelial permeability plays critical roles in vascular functions. The integrity and complexity of intercellular junctions, including tight junctions and adherens junctions, regulate endothelial permeability. Permeability enhancers, such as vascular endothelial growth factor (VEGF), cause cell contraction and junction disruption resulting in intercellular gaps and increased permeability[21].

Hydrogen sulfide (H_2_S), as a gaseous signaling molecule, has been demonstrated to be beneficial for a range of cardiovascular diseases, including peripheral and cardiac ischemia [6], [24], [25], atherosclerosis[30]. Although we know that H_2_S is important for cardiovascular health, its effects on endothelial barrier function remain unclear. A few studies investigated the role of H_2_S on hyperpermeability induced by particulate matter in the lung vasculature and ischemia/reperfusion injury in brain microvessels [14], [19], [41]. Yet the complexity of H_2_S metabolism has not been taken into consideration.

In endothelial cells, cystathionine γ-lyase (CSE) is considered to be a predominant source of H_2_S [40]. As a by-product of the transsulfuration pathway, the level of free sulfide is low (i.e. H_2_S, HS^−^ and S^2−^). However, in the presence of free radicals, metal ions and myeloperoxidase, thiyl radicals can be formed leading to persulfide and polysulfide compound. Others and we have also reported that H_2_S can be mobilized from biochemical reservoirs such as acid labile sulfur (iron sulfur clusters), and sulfane sulfur (e.g. thiosulfide, protein per/polysulfide) [23], [36], [37]. Moreover, increasing evidence suggests that sulfane sulfur serves as an active form of H_2_S that is highly potent and may be responsible for some of H_2_S biological effects [18], [29]. To better understand the effect of H_2_S on endothelial permeability, we examined how exogenous and CSE derived endogenous H_2_S could regulate endothelial permeability.

## 2 Materials and methods

### 2.1 Animals

All mice used in this study were housed in accordance with the National Research Council’s Guide for the Care and Use of Laboratory Animals. 12–20 week old male C57BL/6 J wild-type and CSE knockout mice were used for experiments. All animal studies were approved by the LSU Health-Shreveport institutional animal care and use committee (Protocol Number: P-14-040).

### 2.2 Cell culture

Human umbilical vein endothelial cells (HUVECs) (FC-0044, Lifeline Cell Technology) were maintained in endothelial growth medium (LL-0003, Lifeline Cell Technology) with 10% fetal bovine serum (FBS) at 37 °C. Mouse aortic endothelial cells (MAECs) were isolated from wild-type and CSE knockout mice. Mice were anesthetized with an intraperitoneal injection of ketamine (100 mg/kg) and xylazine (8 mg/kg) and perfused with 5 ml phosphate buffered saline (PBS). Thoracic aortae were dissected and cut into 0.5 mm thick rings. Aortic rings pooled from 3 mice were seeded on Matrigel (354,234, Corning) in a 6-well plate and cultured in endothelial growth medium for 5–7 days for endothelial cells to sprout. After removal of aortic ring, Matrigel was digested by dispase (354,235, Corning). Cells were sorted for endoglin (CD105) (13–1051-85, eBioscience) using Dynabeads M-280 Streptavidin (11205D, Invitrogen) and immortalized using temperature sensitive SV40 large T antigen by retrovirus. Isolated MAECs were maintained in Dulbecco’s Modification of Eagle’s Medium (DMEM) (15–013-CV, Corning) with 10% FBS supplemented with Recombinant mouse interferon gamma (IFN-γ) (50-990-843, Fisher Scientific) at 33 °C. For experiments, MAECs are kept at 37 °C without INF-γ for at least 48 h for inactivation of large T-antigens. Both HUVECs and MAECs were starved overnight with low serum medium before experiments. Transfection was performed using siRNA oligos and lipofectamine 2000 (11,668-019, Thero Fisher Scientific).

### 2.3 In vitro permeability assay

Solute permeability was tested using 6.5 mm transwell inserts with 0.4 µm pore size (07–200-154, Fisher Scientific) as previously described [42]. Transwell inserts were coated with fibronectin. Endothelial cells were trypsinized and reconstituted in medium at the concentration of 40,000 cells/ml. 250 μl cells (10,000 cells) were seeded in fibronectin coated transwell inserts and 1370 μl medium was added to the bottom chamber. Cells were cultured for 36 h without changing medium. At the time of study, 25 μl albumin–fluorescein isothiocyanate (FITC) conjugate (10 mg/ml) (A9771–100MG, Sigma-Aldrich) with or without sulfide donors was added to inserts. At certain time points, 50 μl medium was collected from bottom chambers with 50 μl fresh medium added back immediately. Sampled medium was diluted and fluorescence was measured at 495 nm/519 nm. For MAECs, phenol red free DMEM (17–205-CV, Corning) was used for transwell permeability assays. For transendothelial electrical resistance (TEER), transwell inserts with endothelial monolayers were transferred to a cell culture plate embedded with chopstick electrodes [11]. Cells were allowed to rest for 20 min before the resistance was measured using EVOM (World Precision Instruments).

### 2.4 In vivo permeability assay

In vivo permeability was examined by the modified Miles assay as previously reported [8]. Evans blue (EB) (100 μl, 1% w/v in normal saline) was delivered by tail vein immediately followed by saline, vascular endothelial growth factor 165 (VEGF 165) (293-VE-010) and sulfide donor injections in the ear pinna. After 30 min, mice were euthanized and perfused with 5 ml PBS. Ears and internal organs were harvested. Samples were kept in microcentrifuge tubes and dried at 95 °C overnight on a heat block. EB were extracted from dried tissue in 500 μl formamide at 55 °C for 2 days. EB concentration was determined by absorbance at 630 nm.

### 2.5 Immunocytochemistry and immunoblotting

For immunocytochemistry (ICC), cells were cultured on a glass coverslip and fixed with 4% formaldehyde and permeabilized using 0.1% Triton X-100. Coverslips were blocked with 10% serum and incubated with primary antibodies for 16 h. After thorough rinsing in Tris buffered saline with 0.1% Tween-20, coverslips were incubated with secondary antibodies for one hour. Images were taken using Nikon NIS Elements. For western blotting (WB), cells were lysed in 2X Laemmli sample buffer and boiled for 5 min. Protein sample was then loaded on 10% SDS denaturing gels and transferred to a PVDF membrane. Nonspecific proteins are blocked with 5% non-fat milk. Proteins of interest are blotted for and appropriate secondary antibodies are used for detection. Antibodies and reagents used can be found in Supplementary Table 1.

### 2.6 Cellular hydrogen sulfide measurement in situ

Free sulfide was measured in cells using a specific fluorescent probe, sulfide fluor-7 acetoxymethylester (SF7-AM) (748,110-1MG, Sigma-Aldrich) [28]. Endothelial cells were incubated with 2.5 μM SF7-AM in phenol red free for 30 min and rinsed with medium. Cells were then treated with sulfide donors. Fluorescence was measured at 495 nm/519 nm. The fluorescent intensity at a certain time point (F0) was divided by fluorescent intensity at zero-time point (Fi), denoted as F0/Fi. Result was presented as the fold change of F0/Fi over control group.

### 2.7 Measurement of hydrogen sulfide metabolites

Sulfide metabolites were measured using HPLC as previously reported (36,37). Briefly, cells were lysed in the reaction buffer (0.1 mM DTPA in 100 mM Tris, pH 9.5) with 0.1% Triton-X-100. For free sulfide, 70 μl sample, 30 μl reaction buffer and 50 μl monobromobimane (MBB) solution (10 mM in acetylnitrile) were mixed in a PCR tube and incubated for 30 min. The reaction was stopped by adding 50 μl stopping solution (200 mM sulfosalicylic acid in H_2_O) followed by 10-minute incubation on ice. For acid labile and sulfane sulfur, 50 μl sample was injected into vacutainers. 450 μl releasing buffer (0.1 mM diethylenetriaminepentaacetic acid [DTPA], 100 mM phosphate buffer pH at 2.6) with or without 1 mM tris(2-carboxyethyl)phosphine (TCEP) were injected to the vacutainers respectively. Tubes were incubated on a rocker for 30 min before the liquid phase was removed using a syringe with a spinal needle. 500 μl reaction buffer was added to absorb the gas phase left in the vacutainer. Eventually, the reaction buffer in the vacutainer was allowed to react with MBB as the free sulfide samples. All steps were performed in a hypoxic chamber (1% oxygen) at room temperature. The fluorescent product sulfide-dibimane (SDB) is analyzed by reverse phase high performance liquid chromatography using an eclipse XDB-C18 (4.6×250 mm) column with gradient elution by 0.1% (v/v) trifluoroacetic acid in acetonitrile.

### 2.8 Cytotoxicity and apoptosis assay

Lactate dehydrogenase (LDH) release was measured in cell culture medium using Pierce LDH Cytotoxicity Assay Kit (88,953, Thermo scientific) according to the manufacture instruction. Cells lysed with detergent (provided in the kit) was considered as 100% release, while vehicle treatment was sat as 0%. Data were presented as a percentage of LDH release. Apoptosis was evaluated by cleaved caspase-3 using western blotting.

### 2.9 Chemicals and reagents

Sulfide donors used in this study include sodium sulfide (Na_2_S) (65,122-06, VWR SCIENTIFIC), GYY4137 (13,345, Cayman Chemical) and diallyl trisulfide (DATS) (10,012,577, Cayman Chemical), sodium disulfide (Na_2_S2), sodium trisulfide (Na_2_S3) and sodium tetrasulfide (Na_2_S4) (SB02-10, SB03-10, SB04-10, Dojindo Molecular Technologies). All donors were freshly prepared in degassed distilled water and diluted with cell culture medium immediately before treatments.

### 2.10 Statistics

Data were reported as mean±standard error of the mean (SEM). *In vitro* experiments were repeated on different days. At least 3 independent experiments were used for statistic analysis. For transwell permeability assay, each independent experiment was performed in replicates for each treatment. Statistical analysis was performed with Graph Pad Prism using Student *t*-test, one-way ANOVA and two-way ANOVA with Tukey post-hoc test. P-values of <0.5 were considered as statistically significant.

## 3 Results

### 3.1 Effects of exogenous H2S on endothelial permeability in vitro

**Fig. 1:**
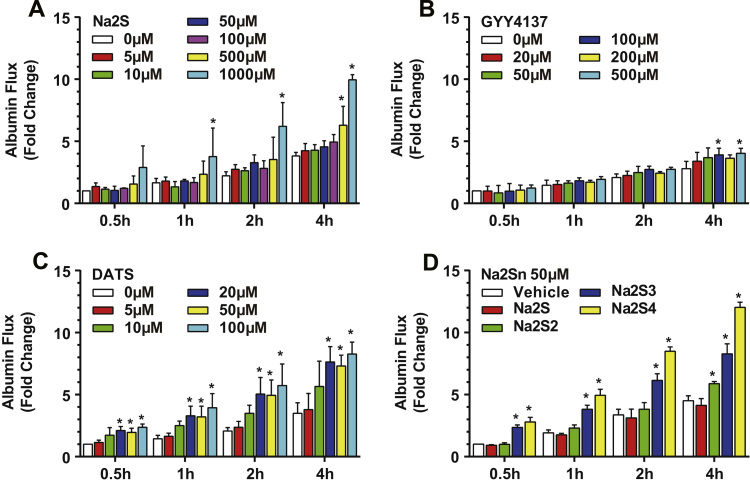
Exogenous hydrogen sulfide increased solute permeability. HUVECs were treated with hydrogen sulfide donors, Na_2_S (A), GYY4137 (B), DATS (C) and inorganic polysulfide donors (D) in transwell inserts at indicated concentrations. FITC-albumin was added to the top chamber and medium was collected at indicated time points over 4 h. * indicates significant difference from vehicle treatment group at the same time point (n=3, p<0.05).

To examine the effect of exogenous H_2_S on endothelial solute permeability, we first measured albumin flux across human umbilical vein endothelial cell (HUVEC) monolayer over 4 h after treatments of two commonly used free sulfide donors, Na_2_S and GYY4137. Both Na_2_S (5 μM–100 μM) and GYY4137 (20 μM–50 μM) had little effect on permeability at low concentrations over a 4-hour time course (Fig. 1A-B). However, Na_2_S at 500 μM and 1 mM concentrations, increased permeability to 6.29±0.88 (p=0.0205) and 9.96±0.24 (p<0.0001) fold respectively although these concentrations are not pathophysiologically relevant (Fig. 1A). Similarly, 100 μM and 500 μM GYY4137 increased albumin flux to 3.90±0.31 (p=0.0461) and 4.04±0.23 fold (p=0.0162) respectively (Fig. 1B). We next tested the polysulfide donor, diallyl trisulfide (DATS). In comparison, 20 μM DATS was able to significantly increase permeability 2.10±0.3 fold quickly within 30 min (p=0.0281). Moreover, 50 μM or 100 μM DATS did not further increase permeability at the 30-minute time point (2.0±0.3 fold, p=0.0395 and 2.4±0.3 fold, p=0.0125). Increased permeability induced by DATS (≥20 μM) was sustained up to 4 h (p<0.05), although no further increase over the control group was observed after 30 min. To rule out potential effects of two allyl groups of DATS, we also performed transwell permeability assays with inorganic polysulfide compounds, Na_2_S_2_, Na_2_S_3_ and Na_2_S_4_. Importantly, the more sulfur atom in the donor molecule, the more potent it increased permeability (Fig. 1D). At the 30-minute time point, 50 μM Na_2_S_3_ increased permeability 2.36±0.19 fold (p=0.0199), producing similar responses as DATS at the same concentration (1.95±0.20, p=0.0395). Therefore, for the rest of this study we used DATS to investigate polysulfide induced permeability.

### 3.2 Measurement of H_2_S metabolites

**Fig. 2:**
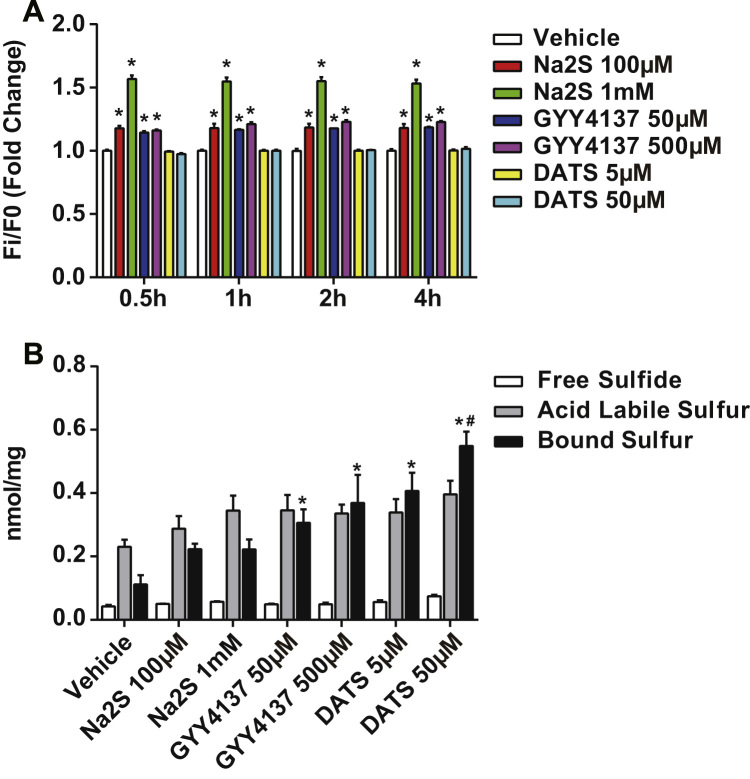
Na_2_S and GYY4137 increased free sulfide while DATS increased bound sulfane sulfur. A: HUVECs were pre-incubated with SF7-AM for 30 min and rinsed with medium. Sulfide donors were given to cells at indicated concentrations. Fluorescent intensity was measured at different time points. * indicates significant difference between vehicle treatment at the same time point (n=6, p<0.05). B: HUVECs were treated with sulfide donors at indicated concentrations. Sulfide pools were measured by the MBB method. * indicates significant difference from vehicle treatment. # indicates significant difference from GYY4137 (50 μM and 500 μM) and DATS 5 μM. N=3–6, p<0.05.

Na_2_S and GYY4137 are both free sulfide donors. Na_2_S releases a bolus amount of H_2_S immediately upon hydration (peaks in seconds with a half-life ~5 min), whereas GYY4137 releases H_2_S gradually and lasts longer (plateaus in minutes and lasts for hours) [26], [32]. Although DATS may generate H_2_S with cellular thiol metabolism, it also serves as a persulfide donor. Thus it was important to distinguish whether changes in intracellular free sulfide or polysulfide were associated with increased endothelial permeability. To examine this, we first measured intracellular free sulfide using a specific fluorescent probe, SF7-AM (Fig. 2A). After 30-minute incubation of HUVECs with Na_2_S at 100 μM and 1 mM, the fluorescence increased 1.2±0.02 and 1.6±0.03 fold (p<0.0001) compared to the vehicle treatment group. GYY4137 (50 μM and 500 μM) also increased fluorescence but to a lesser extent (1.1±0.01 fold, p<0.0001). However, none of the treatments further increased fluorescence intensity after 30 min. Importantly, DATS did not increase SF7-AM signal over a 4-hour time course (Fig. 2A).

Next we performed analytical measurement of defined H_2_S biochemical pools after 4-hour donor treatments in HUVECs using the MBB method (Fig. 2B). Neither free H_2_S nor acid labile sulfur were significantly increased by various donors at 4 h. Conversely, 50 μM DATS, which was most effective at inducing permeability, increased bound sulfane sulfur from 0.112±0.041 to 0.549±0.065 nmol per mg total protein (p<0.0001). 5 μM DATS and GYY4137 (50 μM and 500 μM) also increased bound sulfane sulfur but to a substantially lower amount than 50 μM DATS (p<0.05). These data suggest there is a threshold of polysulfide levels that stimulates increased endothelial solute permeability.

### 3.3 Effects of free sulfide and polysulfide on cell-to-cell junction and actin cytoskeleton

**Fig. 3:**
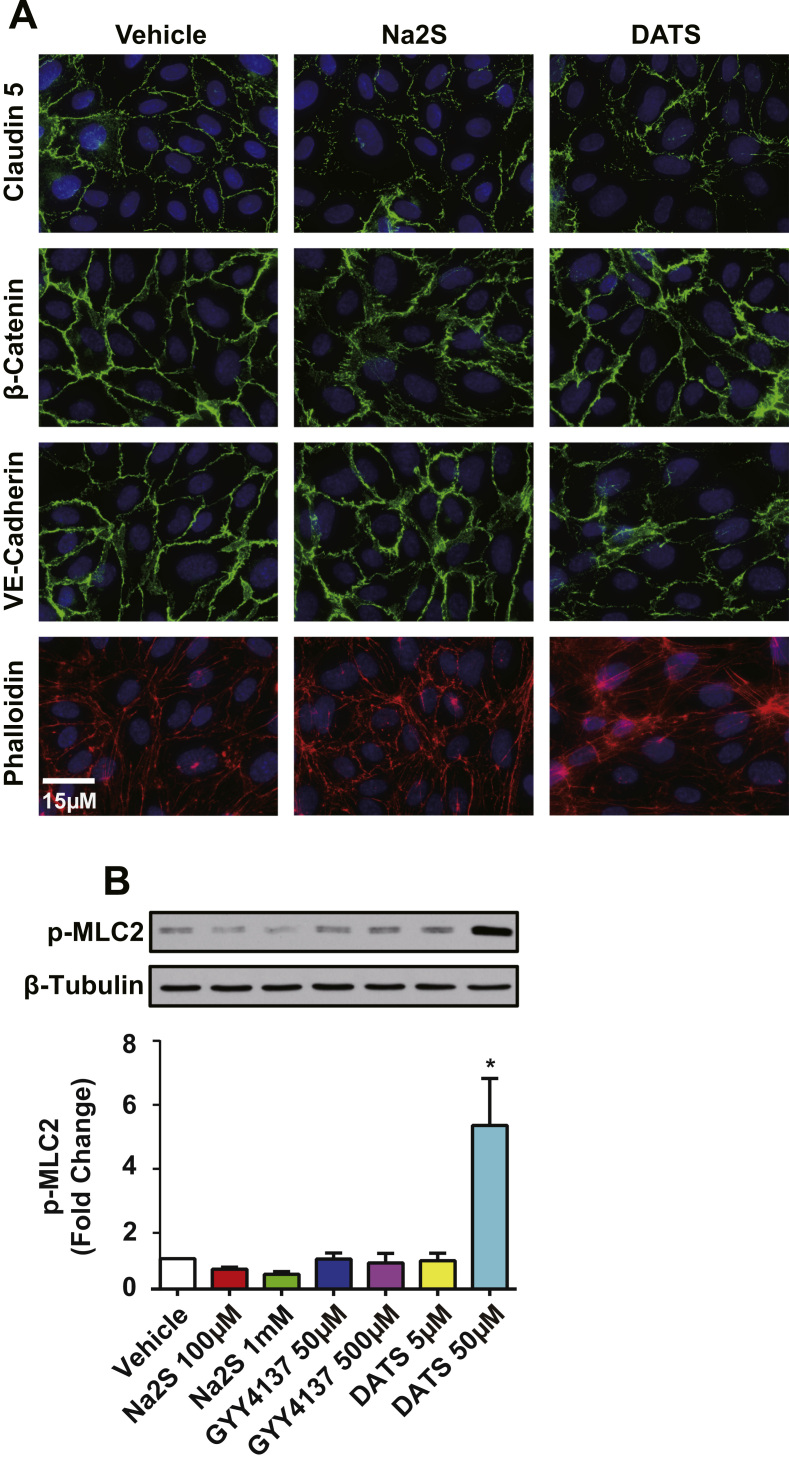
Exogenous sulfide induced remodeling of endothelial cell junctions and actin cytoskeleton. A: Immunofluorescence was performed for junction proteins and actin on HUVECs after 1 h 500 μM Na_2_S or 20 μM DATS. Among the shown images, VE-cadherin and actin (phalloidin) were co-stained for the same cells. B: Illustrative western blots and the quantification for HUVECs treated with Na_2_S, GYY4137 and DATS at indicated concentrations for 1 h. * indicates significant difference from vehicle treatment (n=3, p<0.05).

Increased solute permeability is regulated primarily through cell-to-cell junctions composed of tight and adherens junctions. This pathway is regulated by structural proteins such as claudin-5 at tight junctions and VE-cadherin and β-catenin at adherens junctions. Disruption of these proteins compromises endothelial barrier function. We performed immunostaining for claudin-5, VE-cadherin and β-catenin in HUVECs treated with 500 μM Na_2_S or 20 μM DATS. Continuous strands of claudin-5 and VE-cadherin indicate integrity of tight junctions and adherens junctions, respectively. Conversely, discontinuous and interdigitated junctional protein alignment indicates disruption of cell-to-cell junctions. By comparison, DATS treatment resulted in substantial loss and disruption of claudin 5 and VE cadherin at endothelial junctions (Fig. 3A). Conversely, Na_2_S treatment did not alter endothelial junctional protein localization similar to DATS. Enhanced actin stress fiber formation contributes to increased paracellular permeability and facilitates junction disruption [22]. Interestingly, DATS but not Na_2_S increased actin stress fiber formation as seen by phallodin staining. This is further evidenced by differential effects of sulfide donors on myosin light chain phosphorylation (p-MLC). DATS (50 μM) increased p-MLC 5.4±2.7 fold (p=0.0024) (Fig. 3B); whereas, none of the other donors similarly affected p-MLC. Thus, polysulfide is most potent at disrupting endothelial cell junctions and inducing stress fiber formation.

### 3.4 Effects of free sulfide and polysulfide on cell survival

**Fig. 4:**
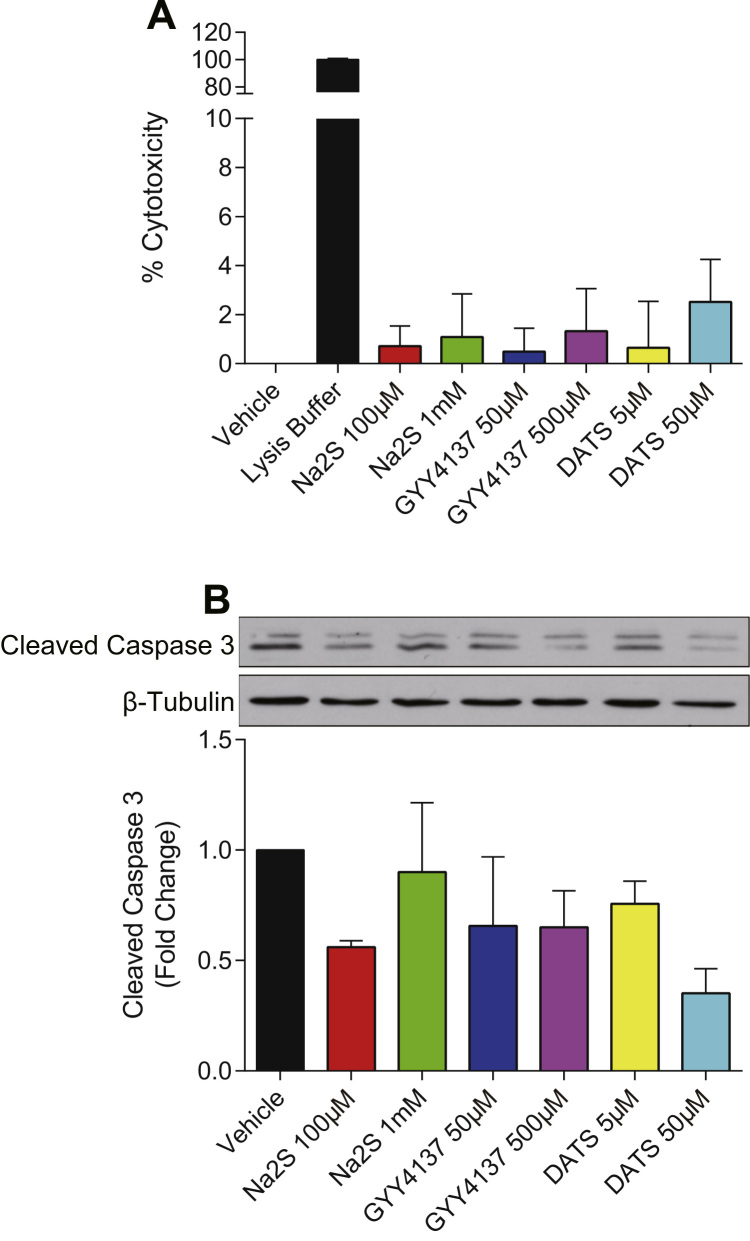
Hydrogen sulfide donors did not affect endothelial cell survival. A: HUVECs were treated with Na_2_S, GYY4137 and DATS at indicated concentrations for 4 h. Medium as taken for LDH activity assay (n=3, p>0.05). B: Illustrative western blots and the quantification for HUVECs treated with sulfide donors for 4 h (n=3, p>0.05).

To determine whether sulfide donor mediated changes in permeability were due to cytotoxicity, we performed the LDH release assay to examine cell viability. HUVECs were treated with Na_2_S, GYY4137 or DATS for 4 h at various doses that did or did not alter permeability. None of the sulfide donor treatments showed significant toxicity within the four-hour study period (p>0.05) (Fig. 4A). Similarly, the level of cleaved caspase 3 was also not increased, indicating that treatments did not induce apoptosis (p>0.05) (Fig. 4B). Therefore, treatment cytotoxicity is not contributing to endothelial solute permeability changes.

### 3.5 Effects of CSE expression on endothelial barrier function

**Fig. 5:**
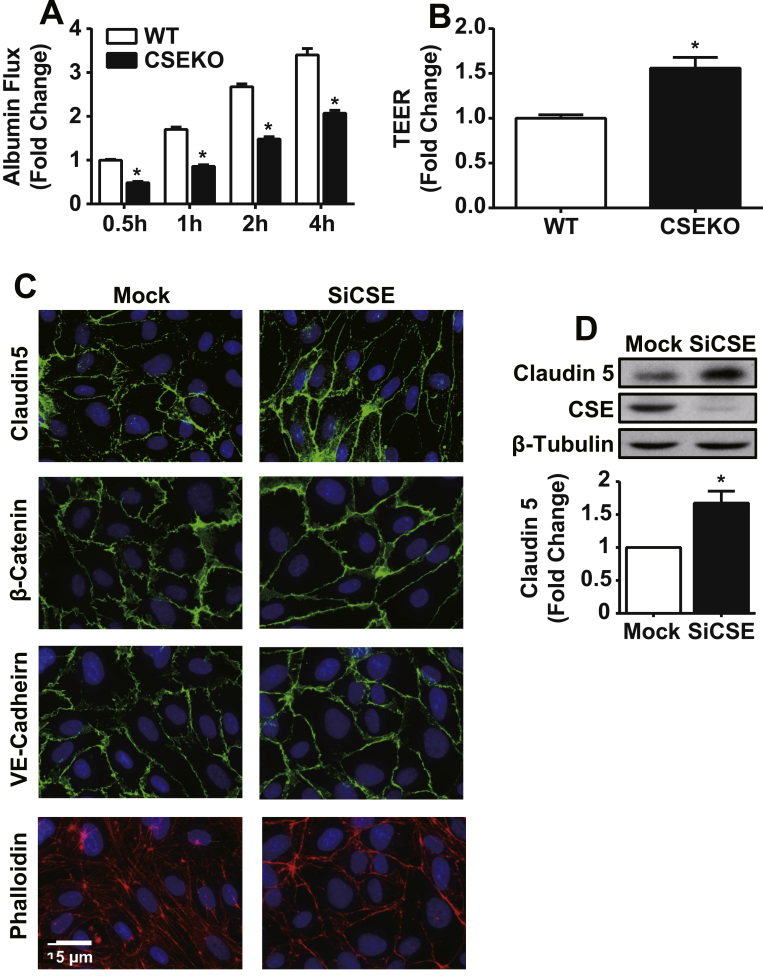
CSE expression increased basal endothelial permeability. MAECs isolated from wildtype and CSE knockout mice and cultured on transwell inserts. A: Solute permeability was assessed by adding FITC-albumin to the top chamber and collecting medium at indicated time points over 4 h. * indicates significant difference between wildtype and CSE knockout MAECs treatment group at the same time point (n=3, p<0.05). B: Transendothelial resistance (TEER) across confluent MAECs on the inserts to determine ion permeability (n=3, p<0.05). CSE expression regulates junction and actin cytoskeleton structure. HUVEC were transfected with siRNA against CSE (siCSE). Mock treated and siCSE transfected cells were cultured to confluence. C: Immunofluorescence was performed for junction proteins and actin. D: Claudin 5 and CSE were analyzed by western blotting after transfection. * n=4, p<0.05.

Next, we examined whether endogenous H_2_S and polysulfide contributes to regulation of endothelial permeability. To investigate the role of CSE, aortic endothelial cells from both wild-type and CSE knockout mice were isolated and used in culture. MAECs isolated from CSE knockout mice had only 60.9±3.5% accumulative albumin flux over 4-hour time course compared to wild-type MAECs (p<0.0001), revealing that CSE knockout MAECs have enhanced barrier function. (Fig. 5A). Similarly, transendothelial electrical resistance (TEER) was found to be 1.56±0.2 fold greater in CSE knockout MAECs compared to wild-type MAECs (p=0.0114), indicating tighter barrier properties (Fig. 5B).

To independently confirm this observation, we performed CSE siRNA knockdown experiments in HUVEC and examined junctional protein and actin cytoskeleton staining. HUVECs with reduced CSE expression revealed greater junction protein alignment of claudin-5, β-catenin, and VE cadherin along with decreased stress fiber formation across the cell (Fig. 5C). Western blotting revealed that claudin-5 expression level was significantly elevated 1.6±0.4 fold in CSE knockdown HUVECs (p=0.0098) (Fig. 5D). These data demonstrate that lack of CSE expression improves junction barrier function by enhancing tight junctions.

### 3.6 Polysulfides regulate permeability in CSE deficient MAECs

**Fig. 6:**
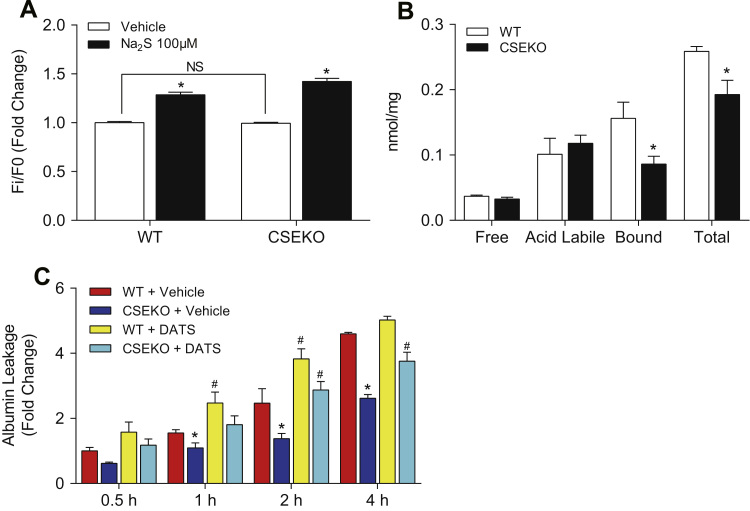
Sulfane sulfur derived from CSE regulates endothelial permeability. A: Free sulfide was measured by SF7-AM. * indicates significant difference from vehicle treatment (n=3–6, p<0.05). B: Sulfide pools were measured by the MBB method. * indicates significant difference between wildtype and CSE knockout MAECs (n=3–6, p<0.05). C: Solute permeability was evaluated by albumin flux. * indicates significant difference between wildtype and CSE knockout MAECs. # indicates significant difference between vehicle and DATS (20 μM) treatments. N=3, p<0.05.

Since CSE maintains a basal level of endothelial permeability, we next wanted to know whether it is free sulfide or polysulfide that mediates this effect. To answer this question, we first measured free sulfide levels in MAECs using SF7-AM. Interestingly, there was not a significant difference in SF7-AM intensity between wild type and CSE knockout MAECs (p=0.7401) (Fig. 6A). Similarly, free H_2_S levels measured by MBB method also did not differ between the two cell types (p=0.2032) (Fig. 6B). However, the level of bound sulfane sulfur was 0.156±0.06 nmol/mg total protein in wild type MAECs and significantly reduced to 0.086±0.03 nmol/mg total protein in CSE knockout MAECs (Fig. 6B). CSE deficiency in MAECs resulted in 44.9±18.8% decrease in the bound sulfane sulfur pool, which contains polysulfides (p=0.0289). To determine whether polysulfide is sufficient to restore endothelial solute permeability, we treated CSE knockout MAECs with DATS. We found 20 μM DATS was sufficient to recover permeability in CSE knockout MAECs to the same level as wild-type MAECs at 2-hour and 4-hour time points (p<0.05) (Fig. 6C). Thus, CSE regulates endothelial permeability through polysulfide formation.

### 3.7 Effect of CSE expression on endothelial permeability in vivo

**Fig. 7:**
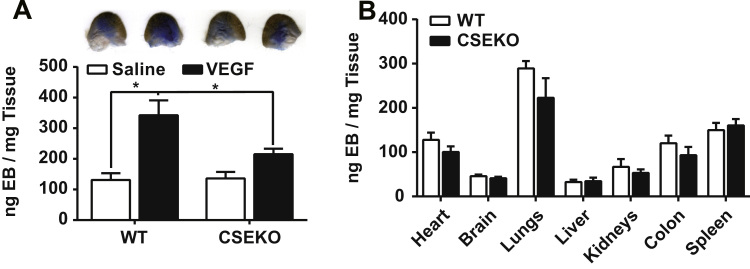
CSE knockout protected mice from VEGF induced hyperpermeability but did not alter basal permeability. Miles assay was performed on wildtype and CSE knockout mice. Tissue were collected after euthanasia, while Evans blue was extracted. A: Saline and VEGF were injected on the ear. * n=4, p<0.05. B: Evans blue was extracted from listed organs to determine basal permeability.

Since CSE critically regulated endothelial solute permeability in vitro, we next examined whether the same effect would be found in vivo. To study permeability in vivo, we performed a Miles assay on the ear pinna with normal saline and VEGF 165 (400 ng per injection, 40 ng/ml) injections. VEGF mediated solute permeability was significantly blunted in CSE knockout mice (215±36 ng EB/mg dry tissue, p=0.0493) compared to wild type mice (342±97 ng EB/mg dry tissue) (Fig. 7A). Potential differences in basal solute permeability using the EB assay was also tested in various organs which were not significantly different between wild type and CSE knockout mice (p>0.05) (Fig. 7B). Thus, in vivo permeability appears to be selectively regulated by CSE.

## 4 Discussion

The regulation of endothelial permeability is important for vascular health. While basal permeability maintains normal solute exchange, vascular hyperpermeability precedes and accompanies a vast array of pathological and physiological events such as inflammation, tumorigenesis, ischemic injury, wound healing and development. The opening of the endothelial paracellular pathway is critical for leukocyte extravasation, which is blocked when junctions are stabilized [35], [39]. Moreover, increased vascular permeability enhances angiogenesis via deposition of extracellular matrix from blood plasma, while reduced permeability is associated with impaired angiogenesis [5], [10], [13]. Therefore, endothelial permeability is fine-tuned in order to adapt to various conditions. However, it was previously not known whether H_2_S regulates endothelial solute permeability and how this may occur.

Studies have reported that H_2_S therapy has protective effects against particulate matter or ischemia/reperfusion induced vascular permeability [14], [41]. However, these studies did not examine roles or mechanisms of H_2_S metabolites on endothelial permeability rather permeability changes were considered as a consequence of pathophysiological events. Additionally, no studies to date have investigated important questions regarding sulfide metabolism and permeability including what sulfur species is biologically active, what endogenous cellular sources are important, and what molecular mechanisms are involved. Our current study provides clear insight into all of these aspects.

To begin with, polysulfides (e.g. DATS), but not Na_2_S or GYY4137, most potently and rapidly increase endothelial permeability in vitro. Although DATS can potentially release free sulfide [34], this process typically requires thiol dependent reduction in the cell [4], [27]. Importantly, our data reveals that DATS and other polysulfides act distinctly different than free sulfide or GYY4137 highlighting key physiological differences between these moieties. Polysulfide species are reactive and can interact with thiols, including small molecular thiols (e.g. glutathione [GSH]) and protein cysteine residues. Moreover, these polysulfide species have similar or greater reactivity than reactive oxygen species (ROS) and are therefore named reactive sulfur species (RSS) [12].

By comparison, neither Na_2_S nor GYY4137 are effective permeability inducers compared to polysulfides. Instead, they only increased permeability at high concentrations. Exposure to high level of H_2_S is known to be toxic by mitochondrial inhibition. But in our studies, the permeability induced by these treatments was not a result of cytotoxicity. This may be due to the short half-life of free H_2_S (~5 min) in an open environment such as cell culture and the fact that high concentrations of free sulfide can lead to polysulfide formation[16], [32].

An important finding of our study is that CSE expression regulates basal endothelial solute permeability. Our group and others have shown sulfide can be stored in three biochemical reservoirs: free sulfide (i.e. H_2_S, HS^−^, S^2−^), acid labile sulfur (e.g. iron sulfur clusters) and sulfane sulfur (e.g. persulfide, polysulfide) [37]. Under certain circumstances, one biological pool may be mobilized. Importantly, one electron oxidation of free sulfide, in the presence of free radicals, ion metals and peroxidases, gives rise to sulfhydryl radical and subsequent RSS. It is worth noting that under basal conditions, endothelial cells have more sulfane sulfur content than free sulfide. Moreover, lack of CSE in endothelial cells significantly reduced sulfane sulfur level rather than free sulfide. This is consistent with the hypothesis of CSE as an enzymatic source of polysulfide [18].

Both exogenous polysulfide and endogenous bound sulfane sulfur derived from CSE regulate endothelial permeability. As previously mentioned, polysulfide may induce post-translational modification on labile cysteine residues. Such modifications often change the conformation and function of target molecules [43]. In our study, it is possible that polysulfide induces junction protein disruption and stress fiber formation via post-translational modification. Potential candidate molecules are suggested in the literature as targets of post-translational modifications. Phosphorylation of β-catenin dissociates it from VE-cadherin and destabilizes endothelial cell-cell junctions [7], [9], [17]. Additionally, S-nitrosation of β-catenin has been reported be important for VEGF induced hyperpermeability [38]. Likewise, the endothelial actin cytoskeleton also critically regulates endothelial permeability. It is possible that actin thiol sulfhydration may also play a role in permeability responses, as sulfide modification of actin leads to increase polymerization [31]. Upstream signaling pathways controlling the arrangement of actin cytoskeleton could also affect junction stability. Phosphorylation of MLC on Thr-18/Ser-19 induces stress fiber formation causing paracellular pores [1], [20], [33]. It is evident that 50 μM but not 5 μM DATS increased MLC phosphorylation. This dose-dependent effect is similar to H_2_O_2_ induced MLC phosphorylation except that DATS derived polysulfide is more effective [44]. Moreover, loss of CSE expression up-regulated claudin-5 expression and enhanced tight junction arrangement highlighting yet another way to modulate endothelial barrier function. Together, these findings reveal a new area of reactive sulfur species regulation of endothelial solute barrier that requires much more investigation.

The physiological importance of sulfide regulating solute barrier function is reflected by defective VEGF mediated permeability in CSE knockout mice. This finding suggests that stimulation of sulfide formation via CSE is likely critical for numerous vascular physiological functions. Consistent with this idea, we recently reported that CSE knockout mice have compromised angiogenesis activity in the hind limb ischemia model [24], [25]. It is well understood that VEGF induced angiogenesis requires disruption of endothelial junctions that facilitates endothelial cell migration and tip cell formation. Thus, these data provide a potential molecular mechanism whereby loss of CSE results in defective VEGF endothelial cell activation, leading to lesser junction alterations and decreased angiogenesis. However, future studies are clearly needed to better understand how CSE and resulting endogenous sulfide metabolites regulate endothelial cell activation responses.

## 5 Conclusions

In summary, our report reveals novel and critically important roles of exogenous sulfide treatments and endogenous CSE dependent regulation of sulfide metabolite formation in regulating endothelial permeability mainly via polysulfide mechanisms. Polysulfides may in turn lead to alterations of small molecular targets including post-translational modification of proteins to regulate endothelial junctions and signaling molecules. Regulation of sulfide metabolism represents a new and important pathway with which to regulate vascular permeability responses.

## References

[1] Adderley S.P., Lawrence C., Madonia E., Olubadewo J.O., Breslin J.W. (2015). Histamine activates p38 MAP kinase and alters local lamellipodia dynamics, reducing endothelial barrier integrity and eliciting central movement of actin fibers. Am. J. Physiol. Cell Physiol..

[2] Aird W.C. (2007). Phenotypic heterogeneity of the endothelium: I. Structure, function, and mechanisms. Circ. Res..

[3] Aird W.C. (2012). Endothelial cell heterogeneity. Cold Spring Harb. Perspect. Med..

[4] Bailey T.S., Zakharov L.N., Pluth M.D. (2014). Understanding hydrogen sulfide storage: probing conditions for sulfide release from hydrodisulfides. J. Am. Chem. Soc..

[5] Bauer P.M., Yu J., Chen Y., Hickey R., Bernatchez P.N., Looft-Wilson R., Huang Y., Giordano F., Stan R.V., Sessa W.C. (2005). Endothelial-specific expression of caveolin-1 impairs microvascular permeability and angiogenesis. Proc. Natl. Acad. Sci. USA.

[6] Bir S.C., Kolluru G.K., McCarthy P., Shen X., Pardue S., Pattillo C.B., Kevil C.G. (2012). Hydrogen sulfide stimulates ischemic vascular remodeling through nitric oxide synthase and nitrite reduction activity regulating hypoxia-inducible factor-1alpha and vascular endothelial growth factor-dependent angiogenesis. J. Am. Heart Assoc..

[7] Biswas P., Canosa S., Schoenfeld D., Schoenfeld J., Li P., Cheas L.C., Zhang J., Cordova A., Sumpio B., Madri J.A. (2006). PECAM-1 affects GSK-3beta-mediated beta-catenin phosphorylation and degradation. Am. J. Pathol..

[8] Blaschuk O.W., Oshima T., Gour B.J., Symonds J.M., Park J.H., Kevil C.G., Trocha S.D., Michaud S., Okayama N., Elrod J.W., Alexander J.S., Sasaki M. (2002). Identification of an occludin cell adhesion recognition sequence. Inflammation.

[9] Chen X.L., Nam J.O., Jean C., Lawson C., Walsh C.T., Goka E., Lim S.T., Tomar A., Tancioni I., Uryu S., Guan J.L., Acevedo L.M., Weis S.M., Cheresh D.A., Schlaepfer D.D. (2012). VEGF-induced vascular permeability is mediated by FAK. Dev. Cell.

[10] Christoffersson G., Zang G., Zhuang Z.W., Vagesjo E., Simons M., Phillipson M., Welsh M. (2012). Vascular adaptation to a dysfunctional endothelium as a consequence of Shb deficiency. Angiogenesis.

[11] Cromer W., Jennings M.H., Odaka Y., Mathis J.M., Alexander J.S. (2010). Murine rVEGF164b, an inhibitory VEGF reduces VEGF-A-dependent endothelial proliferation and barrier dysfunction. Microcirculation.

[12] DeLeon E.R., Gao Y., Huang E., Arif M., Arora N., Divietro A., Patel S., Olson K.R. (2016). A case of mistaken identity: are reactive oxygen species actually reactive sulfide species?. Am. J. Physiol. Regul. Integr. Comp. Physiol..

[13] Dvorak H.F., Brown L.F., Detmar M., Dvorak A.M. (1995). Vascular permeability factor/vascular endothelial growth factor, microvascular hyperpermeability, and angiogenesis. Am. J. Pathol..

[14] Geng Y., Li E., Mu Q., Zhang Y., Wei X., Li H., Cheng L., Zhang B. (2015). Hydrogen sulfide inhalation decreases early blood-brain barrier permeability and brain edema induced by cardiac arrest and resuscitation. J. Cereb. Blood Flow. Metab..

[15] Giannotta M., Trani M., Dejana E. (2013). VE-cadherin and endothelial adherens junctions: active guardians of vascular integrity. Dev. Cell.

[16] Greiner R., Palinkas Z., Basell K., Becher D., Antelmann H., Nagy P., Dick T.P. (2013). Polysulfides link H2S to protein thiol oxidation. Antioxid. Redox Signal..

[17] Guo M., Breslin J.W., Wu M.H., Gottardi C.J., Yuan S.Y. (2008). VE-cadherin and beta-catenin binding dynamics during histamine-induced endothelial hyperpermeability. Am. J. Physiol. Cell Physiol..

[18] Ida T., Sawa T., Ihara H., Tsuchiya Y., Watanabe Y., Kumagai Y., Suematsu M., Motohashi H., Fujii S., Matsunaga T., Yamamoto M., Ono K., Devarie-Baez N.O., Xian M., Fukuto J.M., Akaike T. (2014). Reactive cysteine persulfides and S-polythiolation regulate oxidative stress and redox signaling. Proc. Natl. Acad. Sci. USA.

[19] Jiang Z., Li C., Manuel M.L., Yuan S., Kevil C.G., McCarter K.D., Lu W., Sun H. (2015). Role of hydrogen sulfide in early blood-brain barrier disruption following transient focal cerebral ischemia. PLoS One.

[20] Joshi A.D., Dimitropoulou C., Thangjam G., Snead C., Feldman S., Barabutis N., Fulton D., Hou Y., Kumar S., Patel V., Gorshkov B., Verin A.D. (2014). Heat shock protein 90 inhibitors prevent LPS-induced endothelial barrier dysfunction by disrupting RhoA signaling. Am. J. Respir. Cell Mol. Biol..

[21] Kevil C.G., Ohno N., Gute D.C., Okayama N., Robinson S.A., Chaney E., Alexander J.S. (1998). Role of cadherin internalization in hydrogen peroxide-mediated endothelial permeability. Free Radic. Biol. Med..

[22] Kevil C.G., Oshima T., Alexander J.S. (2001). The role of p38 MAP kinase in hydrogen peroxide mediated endothelial solute permeability. Endothelium.

[23] Kimura Y., Toyofuku Y., Koike S., Shibuya N., Nagahara N., Lefer D., Ogasawara Y., Kimura H. (2015). Identification of H2S3 and H2S produced by 3-mercaptopyruvate sulfurtransferase in the brain. Sci. Rep..

[24] King A.L., Polhemus D.J., Bhushan S., Otsuka H., Kondo K., Nicholson C.K., Bradley J.M., Islam K.N., Calvert J.W., Tao Y.X., Dugas T.R., Kelley E.E., Elrod J.W., Huang P.L., Wang R., Lefer D.J. (2014). Hydrogen sulfide cytoprotective signaling is endothelial nitric oxide synthase-nitric oxide dependent. Proc. Natl. Acad. Sci. USA.

[25] Kolluru G.K., Bir S.C., Yuan S., Shen X., Pardue S., Wang R., Kevil C.G. (2015). Cystathionine gamma-lyase regulates arteriogenesis through NO-dependent monocyte recruitment. Cardiovasc. Res..

[26] Li L., Whiteman M., Guan Y.Y., Neo K.L., Cheng Y., Lee S.W., Zhao Y., Baskar R., Tan C.H., Moore P.K. (2008). Characterization of a novel, water-soluble hydrogen sulfide-releasing molecule (GYY4137): new insights into the biology of hydrogen sulfide. Circulation.

[27] Liang D., Wu H., Wong M.W., Huang D. (2015). Diallyl trisulfide is a fast H2S donor, but diallyl disulfide is a slow one: the reaction pathways and intermediates of glutathione with polysulfides. Org. Lett..

[28] Lin V.S., Lippert A.R., Chang C.J. (2013). Cell-trappable fluorescent probes for endogenous hydrogen sulfide signaling and imaging H2O2-dependent H2S production. Proc. Natl. Acad. Sci. USA.

[29] Lu C., Kavalier A., Lukyanov E., Gross S.S. (2013). S-sulfhydration/desulfhydration and S-nitrosylation/denitrosylation: a common paradigm for gasotransmitter signaling by H2S and NO. Methods.

[30] Mani S., Li H., Untereiner A., Wu L., Yang G., Austin R.C., Dickhout J.G., Lhotak S., Meng Q.H., Wang R. (2013). Decreased endogenous production of hydrogen sulfide accelerates atherosclerosis. Circulation.

[31] Mustafa AK, Gadalla MM, Sen N., Kim S., Mu W., Gazi SK, Barrow RK, Yang G., Wang R., Snyder SH. (2009). H_2_S signals through protein S-sulfhydration. Sci. Signal..

[32] Olson K.R. (2012). A practical look at the chemistry and biology of hydrogen sulfide. Antioxid. Redox Signal..

[33] Parker W.H., Qu Z.C., May J.M. (2015). Intracellular Ascorbate Prevents Endothelial Barrier Permeabilization by Thrombin. J. Biol. Chem..

[34] Predmore B.L., Kondo K., Bhushan S., Zlatopolsky M.A., King A.L., Aragon J.P., Grinsfelder D.B., Condit M.E., Lefer D.J. (2012). The polysulfide diallyl trisulfide protects the ischemic myocardium by preservation of endogenous hydrogen sulfide and increasing nitric oxide bioavailability. Am. J. Physiol. Heart Circ. Physiol..

[35] Schulte D., Kuppers V., Dartsch N., Broermann A., Li H., Zarbock A., Kamenyeva O., Kiefer F., Khandoga A., Massberg S., Vestweber D. (2011). Stabilizing the VE-cadherin-catenin complex blocks leukocyte extravasation and vascular permeability. EMBO J..

[36] Shen X., Pattillo C.B., Pardue S., Bir S.C., Wang R., Kevil C.G. (2011). Measurement of plasma hydrogen sulfide in vivo and in vitro. Free Radic. Biol. Med..

[37] Shen X., Peter E.A., Bir S., Wang R., Kevil C.G. (2012). Analytical measurement of discrete hydrogen sulfide pools in biological specimens. Free Radic. Biol. Med..

[38] Thibeault S., Rautureau Y., Oubaha M., Faubert D., Wilkes B.C., Delisle C., Gratton J.P. (2010). S-nitrosylation of beta-catenin by eNOS-derived NO promotes VEGF-induced endothelial cell permeability. Mol. Cell.

[39] Vestweber D. (2012). Relevance of endothelial junctions in leukocyte extravasation and vascular permeability. Ann. N.Y. Acad. Sci..

[40] Wang R. (2012). Physiological implications of hydrogen sulfide: a whiff exploration that blossomed. Physiol. Rev..

[41] Wang T., Wang L., Zaidi S.R., Sammani S., Siegler J., Moreno-Vinasco L., Mathew B., Natarajan V., Garcia J.G. (2012). Hydrogen sulfide attenuates particulate matter-induced human lung endothelial barrier disruption via combined reactive oxygen species scavenging and Akt activation. Am. J. Respir. Cell Mol. Biol..

[42] Yao L., Kolluru G.K., Kevil C.G., Zhang W.W. (2013). Intravascular radiocontrast iodixanol increases permeability of proximal tubule epithelium: a possible mechanism of contrast-induced nephropathy. Vasc. Endovasc. Surg..

[43] Yuan S., Patel R.P., Kevil C.G. (2015). Working with nitric oxide and hydrogen sulfide in biological systems. Am. J. Physiol. Lung Cell. Mol. Physiol..

[44] Zhao Y., Davis H.W. (1998). Hydrogen peroxide-induced cytoskeletal rearrangement in cultured pulmonary endothelial cells. J. Cell. Physiol..

