# Supplementary material for: Hydrogen sulfide metabolism regulates endothelial solute barrier function
Source: Redox Biol. 2016 Aug 11;9:157–66. doi: 10.1016/j.redox.2016.08.004 (PMC4993857; doi:10.1016/j.redox.2016.08.004)
Supplement: Supplementary Table 1 — Supplementary material [file mmc1.docx]

**Supplementary Table 1. Antibodies and reagents used for immunocytochemistry and western blotting**

| Antibody/Reagent | Application | Dilution |
| --- | --- | --- |
| VE-cadherin (sc-6458, Santa Cruz) | ICC | 1:50 |
| β-catenin (sc7199, Santa Cruz) | ICC | 1:50 |
| Claudin 5 (ab15106, Abcam) | ICC | 1:50 |
| Phalloidin (A22283, Invitrogen) | ICC | 1:1000 |
| Donkey Anti Rabit IgG (A21206, Invitrogen) | ICC | 1:1000 |
| Donkey Anti Goat IgG (A11078, Invitrogen) | ICC | 1:1000 |
| β-tubulin (2128, Cell Signaling) | WB | 1:2500 |
| CSE (12217-1-AP, Proteintech) | WB | 1:1000 |
| Claudin 5 (13805-1-AP, Proteintech) | WB | 1:1000 |
| Cleaved Caspase 3 (9664S, Cell Signaling) | WB | 1:1000 |
| p-MLC2 (Thr18/Ser19)  (3674, Cell Signaling) | WB | 1:2000 |
| Peroxidase-conjugated Anti-Rabit IgG  (115-035-062, Jackson ImmunoResearch) | WB | 1:2000 |
